# Dr. Ora Clay Swift

**Published:** 1910-04

**Authors:** 


### OBITUARY

---

Dr. Ora Clay Swift, of Brooklyn, died at his home in that city, from pneumonia, aged 35 years. He was a member of the staff of the Bushwick and Mutual Hospitals. He graduated at the Medical Department of the University of Buffalo in 1900.

